# Separating neuronal activity and systemic low-frequency oscillation related BOLD responses at nodes of the default mode network during resting-state fMRI with multiband excitation echo-planar imaging

**DOI:** 10.3389/fnins.2022.961686

**Published:** 2022-09-21

**Authors:** Atsushi Tachibana, Yoko Ikoma, Yoshiyuki Hirano, Jeff Kershaw, Takayuki Obata

**Affiliations:** ^1^Department of Molecular Imaging and Theranostics, Institute for Quantum Medical Science, National Institutes for Quantum Science and Technology, Chiba, Japan; ^2^Research Center for Child Mental Development, Chiba University, Chiba, Japan; ^3^United Graduate School of Child Development, Osaka University, Kanazawa University, Hamamatsu University School of Medicine, Chiba University, and University of Fukui, Suita, Japan

**Keywords:** resting-state fMRI, default mode network, low-frequency oscillation, multiband EPI, BOLD signal

## Abstract

Functional magnetic resonance imaging (fMRI) evaluates brain activity using blood oxygenation level-dependent (BOLD) contrast. Resting-state fMRI (rsfMRI) examines spontaneous brain function using BOLD in the absence of a task, and the default mode network (DMN) has been identified from that. The DMN is a set of nodes within the brain that appear to be active and in communication when the subject is in an awake resting state. In addition to signal changes related to neural activity, it is thought that the BOLD signal may be affected by systemic low-frequency oscillations (SysLFOs) that are non-neuronal in source and likely propagate throughout the brain to arrive at different regions at different times. However, it may be difficult to distinguish between the response due to neuronal activity and the arrival of a SysLFO in specific regions. Conventional single-shot EPI (Conv) acquisition requires a longish repetition time, but faster image acquisition has recently become possible with multiband excitation EPI (MB). In this study, we evaluated the time-lag between nodes of the DMN using both Conv and MB protocols to determine whether it is possible to distinguish between neuronal activity and SysLFO related responses during rsfMRI. While the Conv protocol data suggested that SysLFOs substantially influence the apparent time-lag of neuronal activity, the MB protocol data implied that the effects of SysLFOs and neuronal activity on the BOLD response may be separated. Using a higher time-resolution acquisition for rsfMRI might help to distinguish neuronal activity induced changes to the BOLD response from those induced by non-neuronal sources.

## Introduction

Brain functional magnetic resonance imaging (fMRI) utilizes blood oxygen level-dependent (BOLD) signal contrast to evaluate the neuronal activity in active areas of the brain (Ogawa et al., 1990). Neuronal activity leads to increased blood flow in specific regions, and the subsequent decrease in deoxy-hemoglobin concentration in the activated region is reflected by an increase in the BOLD signal intensity (Obata et al., 2004; Hirano et al., 2011, 2018). Therefore, the BOLD signal is not a direct measurement of brain activity, but rather a measure of secondary hemodynamic changes triggered by neuronal activation (Buxton et al., 2004; Liu, 2013). In addition to signal changes related to neural activity, the BOLD signal may be affected by global changes in arteriovenous blood flow velocity, cardiac pulsation, and respiration (Chang and Glover, 2009; Chang et al., 2009). Since the effect of those changes on the BOLD response is relatively slow in comparison to neuronal activity-related changes, they are called low-frequency oscillations (LFOs) (Murphy et al., 2013).

Tong et al. (2013) have explored low-frequency contributions to the BOLD signal that they identified as systemic low-frequency oscillations (SysLFOs). SysLFOs are considered to be non-neuronal in source and can be widely observed in the brain using fMRI. Although it is thought that SysLFO signal may derive from Mayer waves, CO2 concentration fluctuations and vasomotions, the detailed source of the signal is still unclear (Nilsson and Aalkjaer, 2003; Wise et al., 2004; Julien, 2006; Rivadulla et al., 2011; Sassaroli et al., 2012; Golestani et al., 2015). Tong et al. (2013) suggested that SysLFOs are endogenous to the cerebral blood flow and, due to the time they take to propagate, arrive at different regions at different times. Furthermore, if SysLFOs travel throughout the body with the blood flow, it is likely that related signals will be detectable at peripheral sites (Tong and Frederick, 2014). An example of this was provided by a study where near infrared spectroscopy (NIRS) and resting-state fMRI (rsfMRI) were simultaneously performed (Erdoğan et al., 2016). After bandpass filtering the data to the range 0.01–0.1 Hz, it was found that changes in oxygenation measured at the fingertip are significantly correlated with the global BOLD signal (GS), which was obtained by averaging over the whole brain excluding white matter (WM) and cerebrospinal fluid (CSF). The same group also proposed a method for estimating the arrival time of a SysLFO in a particular region of the brain. The method estimates the delay between the GS and the BOLD signal at each pixel, and then a SysLFO-MAP of the whole brain is created. SysLFO-MAPs were found to have a significant correlation with the cerebral blood flow measured with dynamic susceptibility contrast (DSC) MRI (Erdoğan et al., 2016; Tong et al., 2017).

Resting-state fMRI is performed while the subject is awake and at rest, so the data reflects the idle state of the brain (Biswal, 2012). It is thought that rsfMRI signals demonstrate the mechanical coupling and connectivity between various regions of the brain. One of the most important networks is the default mode network (DMN), which consists of nodes in the posterior cingulate cortex (PCC), left and right lower parietal lobes (L- and R-IPL), and medial prefrontal cortex (mPFC) (Greicius et al., 2003). The connectivity of neuronal activity is considered to be high if the BOLD signal changes at each of these nodes are highly correlated. Various studies have reported an association between brain diseases, such as dementia and schizophrenia, and DMN connectivity (Greicius et al., 2004; Liang et al., 2006; Barkhof et al., 2014).

Assuming there is some delay in the propagation of neuronal activity throughout the brain, it is likely that there is a time-lag between the different nodes of the DMN. However, it may be difficult to distinguish between the time-lag in the response due to neuronal activity and the arrival of a SysLFO in specific regions. The conventional single-shot EPI (Conv) acquisition protocol widely used for rsfMRI takes about 2,000 ms to image the whole brain. In contrast, multiband EPI (MB) acquisition enables imaging of the entire brain in a much shorter time (TR 500 ms), which means that BOLD signals can be measured with improved time-resolution (Feinberg et al., 2010). MB protocol data therefore contains four times more information about separate neuronal and possible SysLFO-related signals than data acquired with a Conv protocol. Using a higher time-resolution acquisition method might help to distinguish neuronal activity related time-lags from SysLFO delays.

In this study, we evaluated the time-lag between nodes of the DMN using both the Conv and MB protocols to determine whether it is possible to distinguish between neuronal activity and SysLFO related responses during rsfMRI.

## Materials and methods

Eighteen healthy female volunteers (age 26.6 ± 7.1 years) participated in this study. All subjects provided informed consent, and the institutional review board of the National Institutes for Quantum Science and Technology approved the research protocol (# 16-031).

### Data acquisition

All MRI scans were conducted with a clinical 3T MRI system (MAGNETOM Verio 3T; Siemens Healthcare K.K., Erlangen, Germany) equipped with a 32-channel phased-array head matrix coil. A high-resolution T1-weighted sagittal three-dimensional anatomical image was acquired using magnetization prepared rapid-gradient echo (MPRAGE) with the following parameters: TR = 2,300 ms, TE = 1.95 ms, TI = 900 ms, flip angle = 9 degrees, matrix = 512 × 512, FOV = 250 mm × 250 mm, slice thickness = 1 mm, total acquisition time = 4 min 33 s. For rsfMRI scanning, Conv protocol data was acquired with the following parameters: TR = 2,000 ms, TE = 25 ms, flip angle = 90 degrees, matrix = 64 × 64, FOV = 240 mm × 240 mm, slice thickness = 3.8 mm, slice gap = 0.5 mm, repetitions = 204, total acquisition time = 6 min 52 s. The high time-resolution MB protocol rsfMRI data was acquired with a multiband EPI sequence (University of Minnesota sequence CMRR MB EPI VD13A R016a) using the following parameters: TR = 500 ms, TE = 30 ms, flip angle = 44 degrees, matrix = 64 × 64, FOV = 240 mm × 240 mm, slice thickness = 3.8 mm, slice gap = 0.456 mm, multiband factor = 6, repetitions = 600, total acquisition time = 5 min 7 s. All subjects were instructed to lie still and remain awake with their eyes open while watching a red dot on a screen positioned above them. The datasets generated during the current study are available from the corresponding author on reasonable request.

### Preprocessing

Resting-state fMRI data was preprocessed using DPARSF (Version 4.3^1^) and SPM12.^2^ The first 10 images for each subject were discarded to allow the longitudinal magnetization to reach a steady-state. Slice-timing correction and motion correction were both applied. The data was registered and normalized to the Montreal Neurological Institute (MNI) space using the T1 image re-sampled to 3-mm isotropic voxels. Smoothing was performed with a 4 mm FWHM Gaussian kernel. Time courses were filtered for linear trends and then band-pass filtering (0.01–0.1 Hz) was applied. Confounding terms such as head motion, white matter signal and cerebrospinal fluid signal, were regressed out.

### Time-lag analysis

**FIGURE 1:**
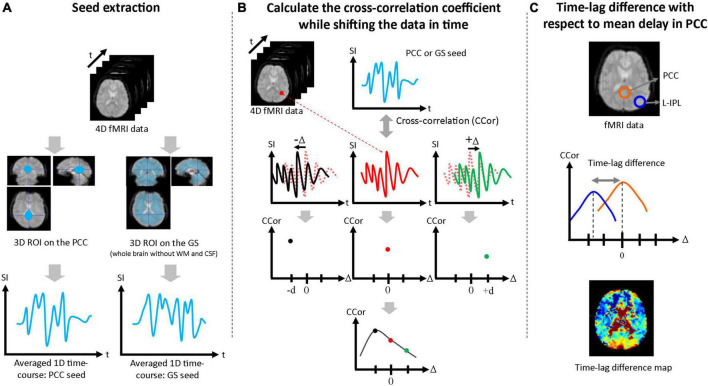
Procedure used to assess time-lags between nodes of the DMN. **(A)** The PCC ROI and GS ROI (whole brain excluding WM and CSF) were extracted and averaged over to generate the 1D seed data. **(B)** The correlation coefficient between the 1D seed and the time-course at each pixel was calculated. Each single-pixel time-course was shifted in time (Δ) and the correlation coefficient was calculated for each value of Δ [i.e., CCor(Δ)]. After cubic spline interpolation with respect to Δ, the maximum value of the correlation coefficient was taken as the time-lag for that pixel. **(C)** Time-lag differences were calculated with respect to the mean time-lag in the PCC ROI and maps were produced to visually evaluate the time-lag across the brain. Mean time-lag differences were also calculated for the mPFC, L-IPL and R-IPL for further analysis.

Seed-based correlation analysis was performed to estimate the time-lags due to functional connectivity of the mPFC, L-IPL, and R-IPL with respect to seed data taken from the PCC and GS. First, a three-dimensional volume seed regions of interest (ROI) was drawn for both the PCC and GS (whole brain without WM and CSF) using the Automated Anatomical Labeling (AAL) brain template, and a one-dimensional seed time-course was produced by averaging over each ROI (Figure 1A). Second, the correlation coefficient was calculated between the one-dimensional seed time-courses and the time-course for each pixel in the brain. The single-pixel time-courses were also shifted in time (Δ), and the correlation coefficient between the seed time-course and the shifted single-pixel time-course at each Δ [i.e., CCor(Δ)] was calculated. The full range of Δ was ± 20 s in 2 s steps for the Conv protocol and ± 5 s in 0.5 s steps for the MB protocol. Cubic spline interpolation was then applied to CCor(Δ) as a function of Δ for each pixel, and the Δ corresponding to the highest value of CCor(Δ) was defined to be the time-lag for that pixel (Figure 1B). To facilitate comparison between the PCC and GS seed results, the mean time-lag in the PCC ROI was calculated and then subtracted from the time-lag of each pixel. Time-lag difference maps were then produced to visually evaluate the time-lag across the brain (Figure 1C), and the mean values in the mPFC, L-IPL, and R-IPL were calculated using ROIs drawn with reference to the AAL brain template. The Wilcoxon signed-rank test was then performed to determine whether the time-lags of each of the DMN nodes with respect to the PCC were significantly different from zero. Bonferroni correction for multiple comparisons was performed by multiplying each *p*-value by 3 for the three different nodes of the DMN.

### Correlation analysis

Spearman’s correlation analysis was performed for the mPFC, L-IPL, and R-IPL to evaluate the relationship between the time-lag of neuronal activity, as represented by the PCC seed analysis, and the time-lag of SysLFOs, as represented by the GS seed analysis. As comparisons were performed for the three different nodes of the DMN, Bonferroni correction for multiple comparisons was applied by multiplying each *p*-value by 3.

## Results

### Time-lag analysis

**FIGURE 2:**
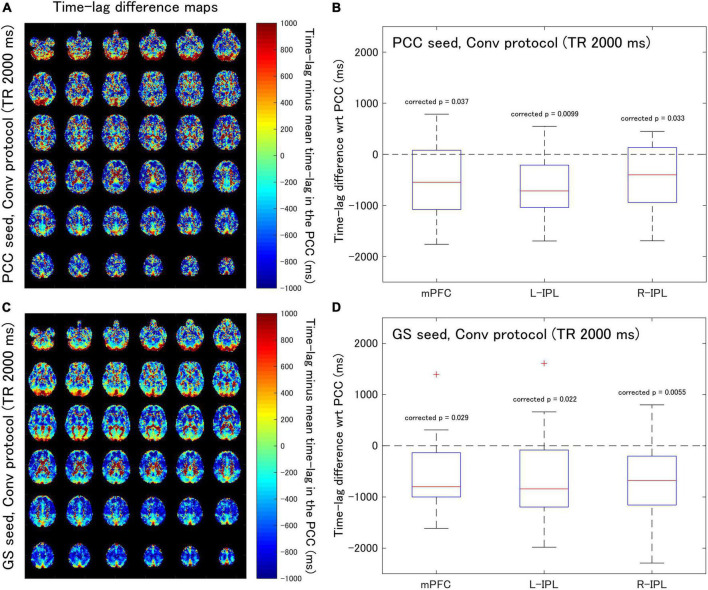
Assessment of the difference in time-lag between the PCC and each of the DMN nodes for data acquired with the Conv protocol (TR = 2,000 ms). Eighteen healthy female volunteers participated in this study. **(A)** Time-lag difference maps calculated using the PCC seed. **(B)** Box-whisker plots of the time-lag difference estimated using the PCC seed for each of the mPFC, L-IPL, and R-IPL ROIs. **(C)** Time-lag difference maps calculated with the GS seed. **(D)** Box-whisker plots of the time-lag difference estimated using the GS seed for each of the mPFC, L-IPL and R-IPL ROIs.

**FIGURE 3:**
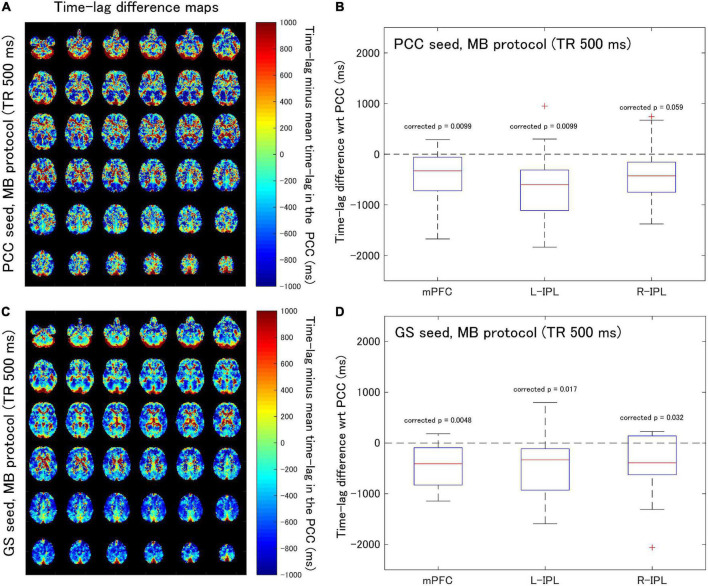
Assessment of the difference in time-lag between the PCC and each of the DMN nodes for data acquired with the MB protocol (TR = 500 ms). Eighteen healthy female volunteers participated in this study. **(A)** Time-lag difference maps calculated using the PCC seed. **(B)** Box-whisker plots of the time-lag difference estimated using the PCC seed for each of the mPFC, L-IPL and R-IPL ROIs. **(C)** Time-lag difference maps calculated with the GS seed. **(D)** Box-whisker plots of the time-lag difference estimated using the GS seed for each of the mPFC, L-IPL and R-IPL ROIs.

Supplementary Figure 1 contains the cross-correlation curves and maps at each Δ for one of the subjects (No. 7). Subsequently, time-lag difference maps were generated (Figures 2A,C, 3A,C), and time-lag analysis was performed on the Conv and MB protocol data for both the PCC and GS seeds.

For the Conv protocol data (Figure 2), the median time-lags estimated with the PCC seed for the mPFC, L-IPL, and R-IPL ROIs differed from the time-lag of the PCC ROI by –547.7, –716.4, and –401.8 ms, respectively (Figure 2B). These differences in time-lag all differed significantly from zero (mPFC, corrected *p* = 0.037; L-IPL, corrected *p* = 0.0099; R-IPL, corrected *p* = 0.033), indicating that the responses were advanced in time compared to that of the PCC. Additionally, the median time-lags estimated using the GS seed differed from that of the PCC ROI by –799.1, –841.6, and –678.7 ms for the mPFC, L-IPL, and R-IPL ROIs, respectively (Figure 2D). The response in each ROI was advanced in time with respect to the PCC because the differences were all significantly different from zero (mPFC, corrected *p* = 0.029; L-IPL, corrected *p* = 0.022; R-IPL, corrected *p* = 0.0055).

When time-lag analysis was performed on the MB protocol data (Figure 3), the median time-lags estimated with the PCC seed differed from the time-lag of the PCC ROI by –329.3, –602.4, and –427.8 ms for the mPFC, L-IPL, and R-IPL ROIs, respectively (Figure 3B). The differences for the mPFC (corrected *p* = 0.0099) and L-IPL (corrected *p* = 0.0099) were significantly different from zero, implying a faster response in comparison to the PCC for those ROIs. However, the response in the R-IPL did not significantly differ from that of the PCC (corrected *p* = 0.059). After analysing the data using the GS seed, it was found that the median time-lags of the mPFC, L-IPL, and R-IPL ROIs differed from the time-lag of the PCC ROI by –408.5, –331.2, and –390.6 ms, respectively (Figure 3D). These results were all significantly different from zero (mPFC, corrected *p* = 0.0048; L-IPL, corrected *p* = 0.017; R-IPL, corrected *p* = 0.032), so the responses were advanced in time compared to the PCC.

### Correlation analysis

**FIGURE 4:**
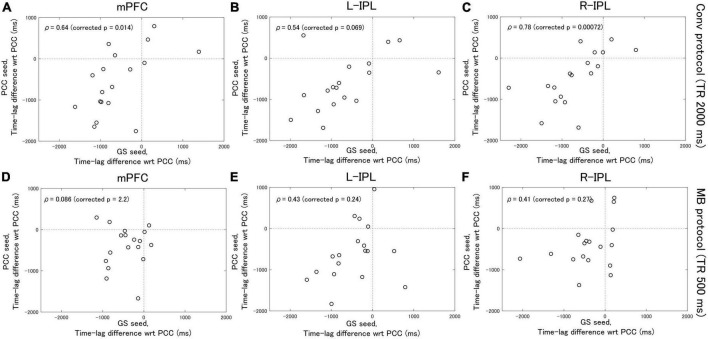
Spearman’s correlation analysis between the time-lag differences calculated with the PCC seed and those calculated with the GS seed. Eighteen healthy female volunteers participated in this study. **(A–C)** Results calculated from the Conv protocol data. **(D–F)** Results calculated from the MB protocol data. The correlations obtained for the MB protocol data were considerably weaker than for the Conv protocol data.

Analysis of the Conv protocol data found substantial correlations between the time-lag differences calculated with the PCC seed (see Figure 2B) and those calculated with the GS seed (see Figure 2D). Spearman’s correlation coefficient for the mPFC, L-IPL, and R-IPL was 0.64 (corrected *p* = 0.014, Figure 4A), 0.54 (corrected *p* = 0.069, Figure 4B), and 0.78 (corrected *p* = 0.00072, Figure 4C), respectively. In contrast, applying a similar analysis to the MB protocol data found that the correlation between the time-lag differences calculated with the PCC (see Figure 3B) and GS (see Figure 3D) seeds was much weaker. Spearman’s correlation coefficient for the mPFC, L-IPL, and R-IPL was 0.086 (corrected *p* = 2.2, Figure 4D), 0.43 (corrected *p* = 0.24, Figure 4E), and 0.41 (corrected *p* = 0.27, Figure 4F), respectively.

## Discussion

The time-lag analysis results indicate that, even though the acquisition sampling rate of the Conv protocol is four times less than that of the MB protocol, a time-lag between nodes of the DMN was detectable with both protocols. In addition, the presence of significant time-lags between nodes indicates that the response in different nodes is highly correlated, but not simultaneous.

Based on the results of previous studies (Biswal, 2012; Erdoğan et al., 2016), in this manuscript the PCC and GS seeds are taken to represent the response due to neuronal activity and SysLFOs, respectively. The time-lag analysis with the PCC seed therefore provides an estimate of the delay in neuronal activity between the PCC and the other nodes of the DMN. Similarly, the time-lag analysis with the GS seed provides an estimate of the delay between the response in different regions of the brain due to SysLFOs. Correlation analysis was then performed to compare the delays estimated with each seed. The fact that significant correlations between the delays are found for the Conv protocol data indicates that the arrival of the response due to neuronal activity cannot be distinguished from the arrival of the response due to SysLFOs. That is, functional connectivity evaluated from Conv protocol data may be heavily influenced by SysLFO signals that are of non-neuronal origin.

On the other hand, the correlation coefficients calculated from the MB protocol data suggest that the arrival of the response due to neuronal activity can be distinguished from the arrival of the response due to SysLFOs in the frequency domain 0.01–0.1 Hz. That is, the higher sampling rate of the MB protocol may allow the SysLFO contribution to the signal to be isolated. Therefore, with suitable adjustments to the analysis procedures, multiband EPI acquisition of rsfMRI data may permit resting-state neuronal activity to be studied without the obfuscating non-neuronal effects of SysLFOs.

There a number of limitations that could have affected the results of this study. First, a relatively high multiband factor of six was employed when acquiring the MB protocol data. With such a high factor it is possible that there was signal leakage from one slice into another simultaneously excited slice (Todd et al., 2016), and this may have influenced the results of the time-lag and correlation analysis. Experiments trialing multiple multiband factors and TR protocols would be required to determine whether the leakage has a significant effect on the results. Second, even though standard motion correction, registration and normalization were applied to the data, it is impossible to guarantee that imperfect correction has not affected the results to some degree. In a similar way, although the data was bandpass filtered, it is not possible to rule out some aliasing of high frequency BOLD signal into the filtered data as noise that influences the results.

## Conclusion

In conclusion, a time-lag between nodes of the DMN was detectable with both the Conv and MB acquisition protocols, with the responses in the mPFC, L-IPL, and R-IPL being temporally advanced with respect to that in the PCC. Correlation analysis of the Conv protocol data suggested that SysLFOs substantially influence the apparent time-lag of neuronal activity. However, correlation analysis of the MB protocol data implied that the effects of SysLFOs and neuronal activity on the BOLD response may be separated. Therefore, using a higher time-resolution acquisition method for rsfMRI might help to distinguish neuronal activity induced changes to the BOLD response from those induced by SysLFOs.

## Data availability statement

The raw data supporting the conclusions of this article will be made available by the authors, without undue reservation.

## Ethics statement

The studies involving human participants were reviewed and approved by the Institutional Review Board of the National Institutes for Quantum Science and Technology (research protocol # 16-031). The patients/participants provided their written informed consent to participate in this study.

## Author contributions

All authors contributed to the conception, design, analysis, and interpretation of the data as well as to drafting the manuscript and revising it critically and read and approved the final version of the manuscript.

## Footnotes

1
http://restfmri.net/forum/DPARSF

2
https://www.fil.ion.ucl.ac.uk/spm/
