# Supplementary material for: Separating neuronal activity and systemic low-frequency oscillation related BOLD responses at nodes of the default mode network during resting-state fMRI with multiband excitation echo-planar imaging
Source: Front Neurosci. 2022 Sep 21;16:961686. doi: 10.3389/fnins.2022.961686 (PMC9534563; doi:10.3389/fnins.2022.961686)
Supplement: Supplementary file 1 [file Image_1.pdf]

Supplementary Material

Supplementary Figures

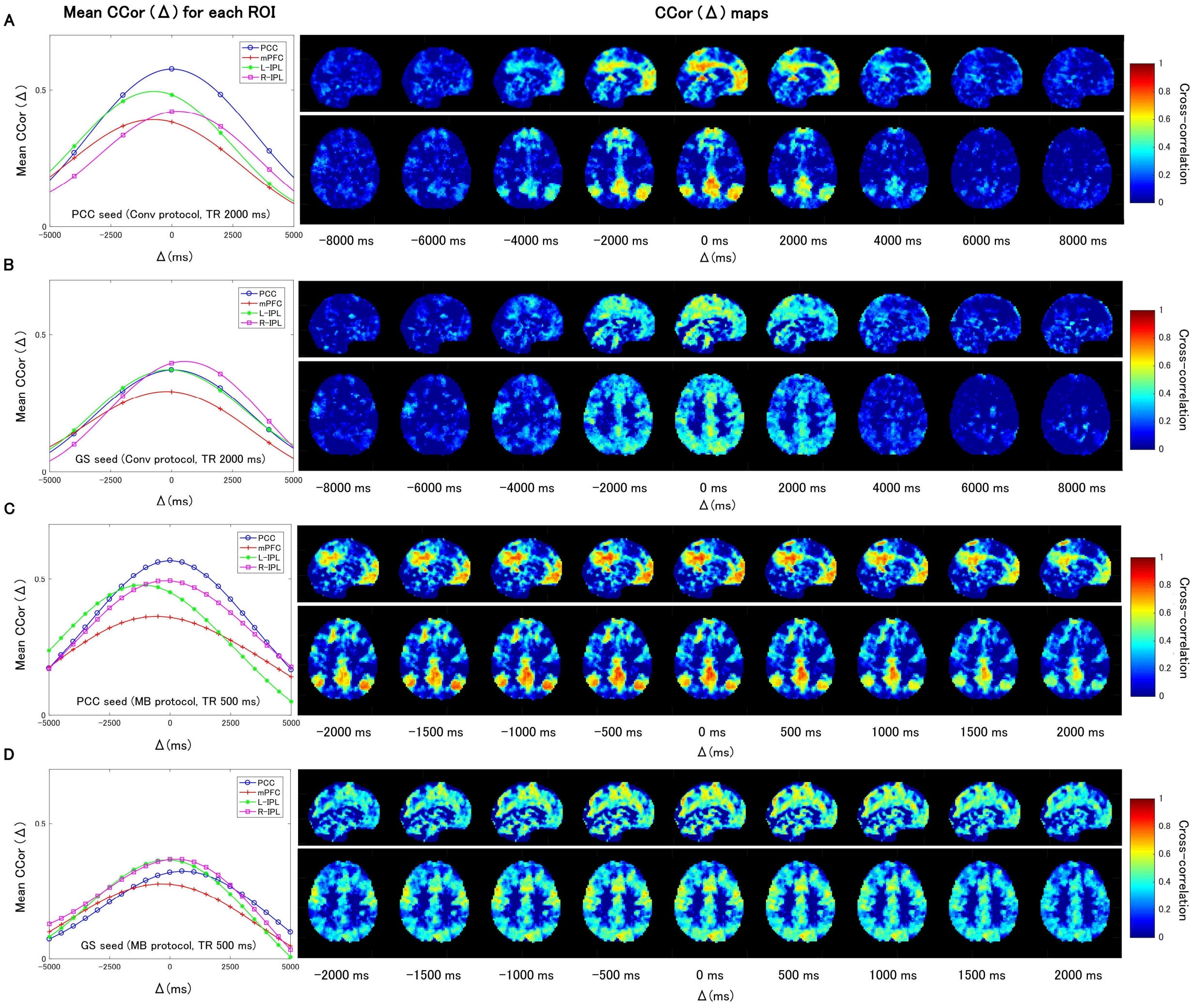

Supplementary Figure 1. Cross-correlation results corresponding to a representative subject (No.7). Maps of CCor(Δ) for each value of Δ are shown on the right with curves corresponding to the mean value in each of the PCC, mPFC, L-IPL and R-IPL ROIs shown on the left. (A) CCor(Δ) calculated from the Conv protocol data using the PCC seed. (B) CCor(Δ) calculated from the Conv protocol data using the GS seed. (C) CCor(Δ) calculated from the MB protocol data using the PCC seed. (D) CCor(Δ) calculated from the MB protocol data using the GS seed.
